# Disability, Work Absenteeism, Sickness Benefits, and Cancer in Selected European OECD Countries—Forecasts to 2020

**DOI:** 10.3389/fpubh.2017.00023

**Published:** 2017-02-27

**Authors:** Mihajlo Jakovljevic, Christina Malmose-Stapelfeldt, Olivera Milovanovic, Nemanja Rancic, Dubravko Bokonjic

**Affiliations:** ^1^Health Economics and Pharmacoeconomics, Faculty of Medical Sciences, University of Kragujevac, Kragujevac, Serbia; ^2^Department of Public Health, The University of Aarhus, Aarhus, Denmark; ^3^Faculty of Medical Sciences, Department of Pharmacy, University of Kragujevac, Kragujevac, Serbia; ^4^Faculty of Medicine of the Military Medical Academy, University of Defence, Belgrade, Serbia

**Keywords:** disability, cancer, sickness benefit, work, absenteeism, Europe, OECD

## Abstract

**Background:**

Disability either due to illness, aging, or both causes remains an essential contributor shaping European labor markets. Ability of modern day welfare states to compensate an impaired work ability and absenteeism arising from incapacity is very diverse. The aims of this study were to establish and explain intercountry differences among selected European OECD countries and to provide forecasts of future work absenteeism and expenditures on wage replacement benefits.

**Methods:**

Two major public registries, European health for all database and Organization for Economic Co-operation and Development database (OECD Health Data), were coupled to form a joint database on 12 core indicators. These were related to disability, work absenteeism, and sickness benefits in European OECD countries. Time horizon 1989–2013 was observed. Forecasting analysis was done on mean values of all data for each single variable for all observed countries in a single year. Trends were predicted on a selected time horizon based on the mean value, in our case, 7 years up to 2020. For this purpose, ARIMA prediction model was applied, and its significance was assessed using Ljung–Box Q test.

**Results:**

Our forecasts based on ARIMA modeling of available data indicate that up to 2020, most European countries will experience downfall of absenteeism from work due to illness. The number of citizens receiving social/disability benefits and the number being compensated due to health-related absence from work will decline. As opposed to these trends, cancer morbidity may become the top ranked disability driver as hospital discharge diagnoses. Concerning development is the anticipated bold growth of hospital discharge frequencies due to cancer across the region. This effectively means that part of these savings on social support expenditure shall effectively be spent to combat strong cancer morbidity as the major driver of disability.

**Conclusion:**

We have clearly growing work load for the national health systems attributable to the clinical oncology acting as the major disability contributor. This effectively means that large share of these savings on public expenditure shall effectively be spent to combat strong cancer morbidity. On another side, we have all signs of falling societal responsibility toward the citizens suffering from diverse kinds of incapacity or impaired working ability and independence. Citizens suffering from any of these causes are likely to experience progressively less social support and publicly funded care and work support compared to the golden welfare era of previous decades.

## Introduction

Permanent or temporary medically confirmed disability has increasingly become a matter of public attention throughout Europe (1). Policy makers are more aware of its far-reaching consequences in terms of demand for hospital and long-term home medical care (2). Another concerning issue is labor market participation. In the environment of accelerated population, aging labor force is shrinking in majority of industrialized northern hemisphere nations (3). Thus, there are ever-growing pressure for harsher social policies, extension of working life in both gender, and inclusion of people with a disability into the labor market. Flexible and generous early retirement policies and disability pensions may become something that belonged in the past in a few decades (4). Although there is large number of evidence on these issues forecasts on transnational European trends are far more scarce (5). The authors try to fill the knowledge gap about disability, work absenteeism, and sickness benefits in selected European OECD countries in relation to cancer morbidity and hospital workload. Thus, this study aims to explain intercountry differences among selected European OECD countries and provides forecasts of future work absenteeism and expenditures on wage replacement benefits.

## Materials and Methods

### Dataset

Two major public registries, World Health Organization (WHO) that issued European health for all database (HFA-DB) (6) and Organization for Economic Co-operation and Development database OECD Health Data (OECD Health), were coupled to form a joint database on 12 core indicators (6, 7). These indicators were related to disability, work absenteeism, and sickness benefits in European OECD countries. Time horizon 1989–2013 was adopted.

Forecasting analysis is the process of making predictions of the future based on past and present data and analysis of trends (8). Forecasting analysis was performed based on the available data for selected countries. Countries entered into the analysis differed depending on data availability since the body of comparable transnational evidence has a “Swiss cheese”-shaped, hollow distribution with significant amount of missing data (9). This was the case due to diverse policies of national authorities reporting to the relevant WHO and OECD bodies in a given historical period.

The 12 indicators for this analysis were selected from the complete list of available indicators, because only these ones could be subject to forecasting analysis due to large number of missing values for other indicators. Therefore, countries observed for “absenteeism from work due to illness” indicator (days per employee per year; source: HFA-DB) were Austria, Czech Republic, Hungary, Netherlands, and Slovenia. For indicator number of “people receiving social/disability benefits per 100,000” (source: HFA-DB), observed countries were Austria, Czech Republic, Estonia, Finland, Hungary, Israel, Netherlands, Norway, Slovakia, Sweden, and Switzerland. For indicator “hospital discharges due to cancer” (source: HFA-DB), observed countries were Austria, Czech Republic, Denmark, Estonia, Finland, Greece, Hungary, Italy, Netherlands, Norway, Portugal, Slovenia, Slovakia, Spain, Sweden, and Turkey. For indicator “public expenditure on incapacity%GDP” (disability + sickness benefits; source: OECD Health Data), observed countries were Austria, Czech Republic, France, Hungary, Luxembourg, Netherlands, Slovenia, Sweden, and United Kingdom. For indicator “compensated absence from work due to illness” (source: OECD Health Data), observed countries were Austria, Belgium, Czech Republic, Denmark, Finland, France, Germany, Greece, Ireland, Italy, Luxembourg, Netherlands, Norway, Poland, Portugal, Spain, Sweden, Switzerland, Turkey, and United Kingdom.

### Data Analysis

Forecasting analysis was done on mean values of all data for each single variable for all observed countries in a single year. On the basis of that mean value trend 1989–2013, we predicted on a selected time horizon, in our case 7 years (2014–2020), how this variable is likely to behave in the future. For this purpose, ARIMA prediction modeling was applied, and its significance was assessed using Ljung–Box Q test.^1^ This test says that if “*p*” is greater than 0.05, it means that the model is correctly specified. In our sample, *p* values using Ljung–Box Q test for the five selected variables in the order of appearance are *p* = 0.782, *p* = 0.819, *p* = 0.232, *p* = 0.907, and *p* = 0.353: associated with absenteeism from work due to illness (days per employee per year) (6), people receiving social/disability benefits per 100,000 (6), hospital discharges due to cancer 1989–2013 (6), public expenditure on incapacity%GDP (disability + sickness benefits; OECD Data), and compensated absence from work due to illness (OECD Data), respectively.

In Tables 1 and 2, values of health indicators are shown as medians, and statistical significance between selected countries for each indicator individually was analyzed using non-parametric Kruskal–Wallis test.

**Table 1:** **Median (95% confidence interval) national values of selected indicators per each country for the period 1989–2013 based on health for all database (HFA-DB) source**.

Country (1989–2013)	HFA-DB absenteeism from work due to illness, days per employee per year	HFA-DB people receiving social/disability benefits per 100,000	HFA-DB% of disabled people of working age engaged in regular occupational activity	HFA-DB disability-adjusted life expectancy (World Health Report)	HFA-DB new invalidity/disability cases per 100,000	HFA-DB hospital discharges, all neoplasms per 100,000
Austria	12.4 (11.7–12.6)	4,646.4 (4,441.1–4,925.3)	–	70.0 (67.1–72.6)	322.5 (267.9–333.0)	2,572.8 (2,397.5–2,673.2)
Belgium	7.2 (7.0–7.4)	2,096.0 (2,088.0–2,405.6)	–	71.0 (67.0–72.7)	–	1,243.6 (1,223.7–1,263.3)
Czech Republic	21.1 (18.2–21.3)	5,065.1 (4,812.5–5,200.1)	22.4 (15.7–24.6)	69.0 (64.2–71.4)	436.4 (385.8–450.2)	1,905.9 (1,751.3–1,920.8)
Denmark	8.3 (8.1–8.6)	3,295.3 (3,307.4–3,683.8)	–	70.0 (66.6–72.1)	–	1,886.6 (1,874.1–2,027.0)
Estonia	9.3 (8.8–9.9)	6,794.3 (5,306.4–7,595.7)	22.4 (19.2–26.1)	67.0 (58.3–72.2)	1,257.5 (912.3–1,664.5)	1,136.8 (930.9–1,168.5)
Finland	7.8 (7.6–8.2)	5,401.0 (5,425.1–5,845.9)	3.9 (3.8–4.1)	71.0 (67.1–73.1)	538.2 (508.3–585.1)	2,209.9 (2,067.5–2,317.8)
France	–	441.4 (421.8–451.8)	–	72.0 (67.8–74.1)	–	2,035.7 (1,993.1–2,103.4)
Germany	16.4 (15.3–17.0)	8,116.2 (7,794.8–8,612.6)	4.2 (4.0–4.2)	71.0 (67.2–73.4)	218.3 (224.3–279.1)	2,296.4 (2,176.9–2,348.4)
Greece	–	748.7 (691.1–1,010.1)	–	71.0 (67.4–73.4)	–	1,444.8 (1,368.0–1,638.3)
Hungary	14.9 (13.2–17.3)	6,547.3 (5,641.6–6,551.9)	9.5 (8.2–9.5)	65.0 (60.0–67.9)	420.0 (364.8–481.0)	2,421.8 (1,925.4–2,495.7)
Ireland	–	3,545.1 (3,245.7–3,592.1)	31.2	71.0 (65.3–74.5)	147.5 (128.4–408.3)	822.1 (814.1–838.8)
Israel	4.1 (3.9–4.4)	3,066.6 (2,822.2–3,248.3)	13.5 (11.2–15.5)	72.0 (66.8–75.3)	328.3 (312.0–343.1)	785.2 (732.5–810.3)
Italy	–	2,181.1 (2,056.0–2,633.4)	16.3 (15.0–16.6)	73.0 (68.3–75.6)	860.1 (728.5–902.1)	1,397.2 (1,360.7–1,480.8)
Luxembourg	9.9 (9.2–10.3)	3,090.6 (2,435.1–3,746.1)	–	71.0 (66.5–74.6)	–	1,732.6 (1,659.5–1,969.9)
Netherlands	12.0 (11.9–13.9)	5,655.3 (5,291.3–5,735.3)	33.5 (31.2–36.5)	71.0 (67.2–73.7)	–	947.0 (927.6–977.6)
Norway	17.3 (17.0–18.1)	6,103.5 (5,802.6–6,151.2)	43.1 (35.7–46.3)	71.0 (66.4–73.1)	631.5 (593.6–668.6)	1,756.4 (1,706.3–1,773.5)
Poland	12.3 (8.2–12.0)	8,776.8 (7,908.6–9,176.2)	20.8 (19.5–21.5)	67.0 (62.7–69.6)	157.2 (139.0–229.0)	1,147.5 (1,127.4–1,668.3)
Portugal	13.0 (10.8–14.7)	3,421.1	–	71.0 (65.1–74.3)	–	780.2 (733.9–882.7)
Slovak Republic	30.9 (26.2–37.0)	5,167.6 (4,433.0–5,042.5)	24.1 (9.6–25.6)	67.0 (62.0–69.5)	329.9 (311.6–383.5)	1,571.7 (1,490.5–1,632.2)
Slovenia	13.8 (13.0–14.4)	–	1.0 (0.8–1.2)	69.0 (63.4–73.0)	380.5 (339.4–387.3)	1,652.2 (1,503.5–1,678.3)
Spain	12.9 (7.4–17.7)	497.6 (472.7–525.8)	27.2 (24.7–28.4)	73.0 (68.5–75.9)	364.0 (321.6–416.9)	882.3 (792.1–884.3)
Sweden	20.0 (18.5–22.5)	4,763.5 (4,652.8–5,232.8)	44.0	72.0 (68.6–73.5)	530.5 (415.7–575.3)	1,654.1 (1,562.4–1,770.3)
Switzerland	–	2,847.5 (2,512.5–2,925.1)	41.7	72.0 (68.4–74.6)	305.3 (249.5–307.8)	1,113.2 (1,074.2–1,197.5)
Turkey	3.4 (2.6–4.0)	9,956.7 (9,191.4–10,781.9)	21.7	65.0 (56.9–70.4)	–	351.1 (308.0–491.9)
United Kingdom	1.1 (1.0–1.2)	–	–	70.0 (66.5–72.9)	–	995.5 (966.9–1,015.8)
Kruskal–Wallis test	*p* < 0.001	*p* < 0.001	*p* < 0.001	*p* < 0.001	*p* < 0.001	*p* < 0.001

**Table 2:** **Median (95% confidence interval) national values of selected indicators per each country for the period 1989–2013 based on health for all database (HFA-DB) and Organization for Economic Co-operation and Development (OECD) Health Data sources**.

Country (period 1989–2013)	HFA-DB incidence of cancer per 100,000	HFA-DB prevalence of cancer (%)	OECD—self-reported absence from work due to illness (number of days lost per person per year)	OECD—compensated absence from work due to illness	OECD—public expenditure on incapacity%GDP (disability + sickness benefits)	Disability benefits per 100,000
Austria	453.5 (435.4–455.8)	3.4 (3.2–3.6)	–	12.4 (11.7–12.6)	2.7 (2.5–2.7)	3,466.2 (3,381.9–3,511.9)
Belgium	467.5 (409.4–504.8)	–	–	9.3 (8.4–10.2)	2.7 (2.5–2.8)	4,582.3 (3,805.4–5,342.9)
Czech Republic	601.0 (570.6–674.5)	3.0 (2.7–3.6)	8.3	21.1 (18.2–21.3)	2.3 (2.2–2.4)	4,081.0 (3,877.7–4,184.6)
Denmark	517.0 (518.7–585.2)	3.5 (3.4–3.8)	6.9 (6.4–8.00)	8.4 (8.3–8.8)	3.8 (3.7–4.2)	1,907.7 (281.5–3,607.2)
Estonia	431.7 (406.6–480.9)	2.4 (2.1–2.7)	8.3 (7.4–9.1)	9.3 (8.8–9.9)	1.8 (1.7–2.1)	6,353.6 (5,622.9–7,209.4)
Finland	453.8 (431.7–488.5)	3.0 (2.7–3.5)	8.4 (8.1–8.7)	–	4.1 (4.0–4.5)	4,941.0 (4,697.7–5,092.7)
France	523.6 (429.8–571.4)	1.3 (0.8–1.8)	9.0	8.0 (7.8–8.2)	1.9 (1.8–2.02)	3,945.7 (3,857.9–4,006.9)
Germany	501.8 (474.2–527.0)	1.7 (1.6–1.8)	–	16.4 (15.5–17.1)	2.1 (2.0–2.2)	1,035.5 (1,021.0–1,071.9)
Greece	–	0.9 (0.6–1.2)	14.8	3.6 (3.4–3.7)	0.9 (0.9–1.1)	1,283.3 (1,153.6–1,331.8)
Hungary	805.9 (510.8–778.8)	–	8.3 (2.7–12.9)	14.9 (13.2–17.3)	2.7 (2.5–2.8)	3,793.3 (3,083.4–4,410.3)
Ireland	384.2 (364.9–399.1)	2.5	–	16.9	1.7 (1.6–1.9)	3,384.0 (3,323.7–3,442.7)
Israel	343.8 (317.0–343.8)	1.1 (0.9–1.1)	4.1 (3.8–4.3)	–	2.8 (2.5–2.9)	–
Italy	510.0 (470.5–519.0)	3.0 (2.8–3.4)	5.8 (4.5–7.1)	–	1.8 (1.7–1.9)	1,602.5 (1,563.9–1,639.4)
Luxembourg	423.6 (398.8–429.5)	–	–	11.2 (10.6–11.6)	3.1 (3.0–3.2)	3,576.4 (3,541.8–3,645.8)
Netherlands	493.9 (487.3–565.6)	2.5 (2.3–2.7)	–	12.0 (11.9–13.9)	3.9 (3.8–4.8)	937.7 (802.0–1,235.7)
Norway	498.9 (479.8–524.4)	3.4 (3.1–3.6)	–	17.3 (16.9–18.1)	4.7 (4.9–4.8)	6,169.5 (6,007.3–6,713.6)
Poland	300.8 (281.9–325.6)	–	6.7	–	3.4 (3.3–4.6)	4,095.7 (3,867.6–4,613.4)
Portugal	318.9 (265.6–363.8)	1.9	6.3	–	2.2 (2.2–2.3)	3,769.5 (3,563.2–3,962.0)
Slovak Republic	399.9 (386.8–443.5)	0.4 (0.4–0.4)	–	13.9 (12.4–14.9)	2.0 (1.8–2.1)	3,998.2 (3,713.2–4,367.6)
Slovenia	459.2 (429.1–519.6)	2.4 (2.2–2.9)	12.9	13.8 (13.0–14.4)	2.4 (2.3–2.6)	2,035.6 (1,849.5–2,173.5)
Spain	–	–	9.2	10.5 (8.9–11.0)	2.5 (2.4–2.5)	2,475.7 (2,452.6–2,493.9)
Sweden	530.1 (518.0–563.5)	4.5 (4.3–4.8)	–	13.3 (11.4–15.1)	5.1 (4.9–5.3)	5,176.5 (4,336.8–5,959.3)
Switzerland	463.4 (443.3–468.3)	–	7.7 (6.9–8.6)	–	2.6 (2.4–2.8)	5,000.4 (4,751.3–5,229.2)
Turkey	60.4 (57.4–93.4)	–	3.4 (2.6–4.0)	–	0.2 (0.2–0.2)	860.8 (750.0–947.8)
United Kingdom	489.2 (482.5–505.4)	–	–	7.8 (7.2–8.0)	2.4 (2.4–2.6)	3,308.5 (3,266.5–3,339.1)
Kruskal–Wallis test	*p* < 0.001	*p* < 0.001	*p* < 0.001	*p* < 0.001	*p* < 0.001	*p* < 0.001

## Results

**Figure 1:**
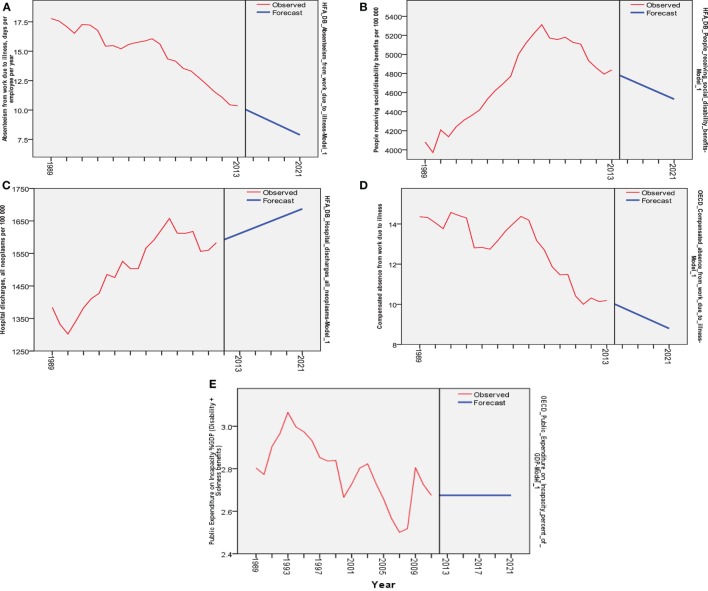
**Forecasting analysis for five selected indicators from 1989 to 2013 based on the past data (mean) for selected countries (red lines represent observed) and forecasting during next 7 years (blue lines represent forecast): (A) decrease in absenteeism from work due to illness, days per employee per year—health for all database (HFA-DB); (B) decrease in people receiving social/disability benefits per 100,000—HFA-DB; (C) increase in hospital discharges due to cancer 1989–2013—HFA-DB; (D) decrease in compensated absence from work due to illness—OECD Health Data; (E) flat trend in public expenditure on incapacity%GDP (disability + sickness benefits)—OECD Health Data**.

Our forecasts based on ARIMA modeling of available data indicate that up to 2020, most European countries will experience a downward trend of absenteeism from work due to illness (Figure 1A) and as will the number of citizens receiving social/disability benefits and compensated absence from work due to illness (Figures 1B,D). Opposed to this, cancer morbidity may become a top ranked disability driver and thereby cause a bold growth in hospital discharges due to cancer (Figure 1C). Public expenditure on incapacity expressed as percentage point share of GDP (disability + sickness benefits observed) remains unknown in current analysis due to large variations and unpredictability (Figure 1E).

More details on each of five prominent indicators (each one reflecting slightly different group of nations) can be found in Figure 1, presenting forecasting analysis with actual data 1989–2013 and time horizon up to 2020. Individual median annual values for all the selected indicators referring to countries observed can be found in Tables 1 and 2, on time horizon 1989–2013.

## Discussion

Of extracted data, we can observe great transnational variability of most indicators depicting disability burden in the European region (10). Significant part of this diversity is attributable to the traditional historical differences in welfare legacies in European geographic regions such as the Western (11), Nordic (12), Mediterranean (13), or Eastern European lands (14).

Work absenteeism due to illness and consecutively number of citizens receiving disability benefits were falling steadily over most of the past three decades and are about to decline further (15). In line with these events, it is anticipated that a contraction of compensated absence from work due to illness as evidenced by OECD Health Data will become reality. In many countries, this is actually driven by policy makers that want to release the pressure on businesses. Employers are obliged to financially compensate the employee’s absence from work caused by sickness (16). The employer’s burden is in many Western European countries shared with the municipal or governmental social support funds (17). Such schemes may serve as inspiration and alternatives to cutting down wage replacement benefits. However, important underlying determinant of societal ability and willingness to invest in and cope with disability-related absence from work is total health expenditure available (18). Evolving landscape of medical spending has some prominently different features in typical Western mature economies and Third World economies among the low-/middle-income nations (19, 20). In many national accounting systems, spending for disability presents a share of the national budget devoted to health care (21). So this actually means that the long-term priority of health in governmental spending will ultimately shape disability/incapacity spending as well. This is applicable to both traditional free market economies and the top ranked emerging BRICS markets as well (22, 23).

The flat line trend forecast for the public expenditure on incapacity should be taken cautiously. It is more realistic to expect downward trend here as well (24).

As previously explained, projected flat trend in public expenditure on incapacity up to 2020 expressed as %GDP share should be taken carefully. This value actually refers to joint disability and sickness benefits among the OECD member nations (25). High level of unpredictable variance leads into suspicion that such trend predicted might not realistically reflect the reality (26). Absenteeism from work due to illness; number of citizens receiving social/disability benefits; and compensated absence from work due to illness are likely (see Figure 1) to downsize in the long run. There are different grounds for this opinion. Some of them might relate to recently published long-run projections of longevity and health expenditures in Eastern Europe (27). Extended life expectancy leads to higher incidence of incapacity cases due to non-communicable illnesses such as dementia (28), traumatism (29), and elderly age itself imposing the need for home care (30). Another indirect sign of medical demand are prescription drugs and associated pharmaceutical spending, which evolves differently in the EU-15 and EU post-2004 members (31). This downsizing of incapacity spending in Europe ultimately means weakened affordability of such social support in many modern day societies. Contemporary governments are threatened by the lack of financial sustainability of current pension and retirement systems (32). Faced with more recent changes such as the global recession and migrant crisis, authorities are tempted to prioritize resource allocation at the expense of persons with a disability (33–35). Actually there is an ongoing opinion that most citizens with moderately impaired working ability should return to work one way or the other (36). Extensive and elaborate social strategies were derived inclusive of gender adjusted perspective. The purpose of these occasionally harsh policies is at least partially to compensate the loss of labor market size due to advanced population aging. Mandatory legal retirement age was moving in Europe from approximately 55 toward 65 in women, while in men, such approach preceded for many years (37).

An increase in hospital discharges due to cancer over the past 25 years presents quite an increase and thereby a significant finding in this study. The prevalence and incidence of most types of malignancies tends to increase in most of Europe (38). This happens due to a variety of reasons: growing citizen expectations (39), earlier diagnostic frontier, medical innovation such as mAbs medicines (40), radiation oncology (41), and imaging diagnostics advances to name just a few (42). Cancer morbidity and its consequences for the national health systems were relevant for this study to study because it is the major driver of disability in most European OECD nations.

Based on all projections, it appears that many European countries may experience shrinking disability-related social costs driven by absenteeism, incapacity benefits, and absence compensations from public funds. This trend is not in line with ongoing morbidity developments since the global burden of disease continues to grow further even in industrialized European countries (43). This fact reflects itself to the social burden of disability caused by illness expressed in (disability-adjusted life year unit), which was applied by the Global Burden of Disease Project (44). These major risk factors contributing to disability were identified to a great extent and determined on national and regional level within the comparative risk assessment framework (45). In its essence, cancer morbidity appears to remain one of the top drivers of permanent or long-lasting disability (46, 47). This is the core reason why we included hospital discharges due to cancer as the observed indicator of national health expenditure caused by malignant neoplasms. Thus, we were capable to reveal long-term hidden morbidity trend that is going to shape social burden of incapacity in the future of Europe (48).

## Conclusion

What we might be able to see here at a number of European OECD nation states presents a contradiction to a certain extent and a great challenge. We have clearly growing work load for the national health systems attributable to the clinical oncology acting as the major disability contributor. This effectively means that large share of these savings on public expenditure shall effectively be spent to combat strong cancer morbidity (49). Some of the possible strategies to tackle these challenges are heavier investment into the preventive public health interventions and early screening detection of cancer. In return, strengthening efficiency of preventive and clinical interventions should make relief on absenteeism and disability costs attributable to late diagnosed, advanced stage cancer (50).

On another side, we have all signs of falling societal responsibility toward the citizens suffering from diverse kinds of incapacity or impaired working ability and independence (51). Regardless of malignant tumors, incapacity is driven to a large extent by diabetes, COPD (52), traffic and other traumatism (53), depression (54), or addiction disorders (55). Citizens suffering from any of these causes are likely to experience progressively less social support and publicly funded care and work support compared to the golden welfare era of previous decades (56). This challenge will remain on top of agenda of policymakers in OECD and developing countries alike. Such a concerning uneasy future is caused by a variety of global socioeconomic developments worldwide. However, impact of population aging shapes the landscape. It implies necessity of labor markets to adapt from the historical demographic growth model toward shrinking demographic pyramid of “silver tsunami” (57). How much contemporary societies will achieve cost-effective solutions to the problem of inclusion and support of disabled citizens yet remains to be seen (58). Provided insights into the forthcoming legislative developments up to 2020 in European OECD countries should be an impulse toward more ambitious research particularly the one targeted toward leading emerging markets of tomorrow.

## Author Contributions

All authors listed, MJ, CM-S, OM, NR, and DB, have made substantial, direct, and intellectual contribution to the work and approved it for publication.

## Conflict of Interest Statement

The authors declare that the research was conducted in the absence of any commercial or financial relationships that could be construed as a potential conflict of interest.

## Footnotes

^1^*Ljung-Box Statistics for ARIMA Residuals in R*. Available from: http://stats.stackexchange.com/questions/64711/ljung-box-statistics-for-arima-residuals-in-r-confusing-test-results.
